# The Association between Unhealthy Food Consumption and Impaired Glucose Metabolism among Adults with Overweight or Obesity: A Cross-Sectional Analysis of the Indonesian Population

**DOI:** 10.1155/2023/2885769

**Published:** 2023-03-22

**Authors:** Adriyan Pramono, Deny Y. Fitranti, K. Heri Nugroho, M. Ali Sobirin, Ahmad Syauqy

**Affiliations:** ^1^Department of Nutrition, Faculty of Medicine, Diponegoro University, Tembalang, Semarang 50275, Indonesia; ^2^Center of Nutrition Research (Cenure), Diponegoro University, Tembalang, Semarang 50275, Indonesia; ^3^Department of Internal Medicine, Faculty of Medicine, Diponegoro University, Tembalang, Semarang 50275, Indonesia; ^4^Department of Pharmacology and Therapeutics, Faculty of Medicine, Diponegoro University, Tembalang, Semarang 50275, Indonesia; ^5^Department of Cardiology and Vascular Medicine, Faculty of Medicine, Diponegoro University, Tembalang, Semarang 50275, Indonesia

## Abstract

**Background:**

It has been shown that dietary patterns are associated with glucose control. However, the association between the types of food consumed and blood glucose in overweight or obese individuals is still unclear. The present study aimed to determine the association between unhealthy food consumption and impaired glucose metabolism in adults with overweight or obesity.

**Methods:**

The analysis presented in this study was based on the data from a population-based, cross-sectional, nationally representative survey (Indonesian Basic Health Research 2018/RISKESDAS 2018). The body mass index (BMI) was calculated as weight (kg)/height squared (m^2^) and was determined based on the World Health Organization (WHO) criteria for the Asian population. A validated questionnaire and food card were used to assess the diet. Fasting plasma glucose and 2-hpost-prandial glucose were employed to determine blood glucose markers.

**Results:**

In total, 8752 adults with overweight or obesity were included in this analysis. We found that consumption of sweet, grilled, and processed foods was associated with impaired fasting plasma glucose (IFG) before and after adjustment (*p* < 0.05). Consumption of high-fat foods was also associated with impaired glucose tolerance (IGT) for all models tested (*p* < 0.05). Furthermore, all models showed a link between processed food consumption and combined glucose intolerance (CGI) (*p* ≤ 0.001).

**Conclusions:**

Differential food group consumption was associated with IFG, IGT, and CGI in Indonesian adults who were overweight or obese.

## 1. Introduction

Various epidemiological studies indicate a trend toward an increase in the prevalence of type 2 diabetes (T2D) worldwide. Furthermore, the World Health Organization (WHO) predicts that 350 million people will have T2D in the forthcoming years [1]. Type 2 diabetes prevalence has increased sharply in developed and developing countries [2, 3]. Interestingly, the prevalence of T2D in Southeast Asia is considerably higher than in other developed countries. More specifically, a recent study indicates that Indonesia is among the countries with a high prevalence of T2D [4].

A prediabetes condition often initiates type 2 diabetes. Prediabetes is defined as a condition with impaired glucose tolerance (IGT), defined by a 2-h glucose concentration between 140 and 199 mg/dl and/or an impaired fasting plasma glucose (IFG) level (i. e., between 100 and 125 mg/dl) [5]. A recent review describes that individuals with IFG mainly show hepatic insulin resistance and normal or slightly lower whole-body insulin sensitivity. In contrast, individuals with IGT have normal to slightly reduced hepatic insulin sensitivity and show moderate to severe reduced skeletal muscle insulin sensitivity [6]. Insulin resistance contributes to hyperglycemia and hyperlipidemia, all risk factors for developing T2D and cardiovascular diseases (CVDs) [7].

Dietary pattern management is an essential determinant of blood glucose control, even in people without T2D [8]. According to a recent study on the Iranian population, long-term healthy diet quality is associated with a lower risk of CVDs [9]. In addition, a study on China's population also showed that high salt intake was associated with T2D [10]. Among US adults, increased consumption of added sugar worsens the risk of CVD death in a dose-dependent manner [11]. Moreover, among men and women in the US, fried food consumption was associated with T2D and CVDs. More interestingly, the association was mediated by obesity status in that study [12].

In addition, the findings regarding the relationship between fruit consumption and metabolic disease are also inconsistent. Meta-analyses of randomized controlled trials showed that fruit juice consumption was not associated with T2D risk [13]. However, fruit consumption (whole fruit) was associated with a lower incidence of T2D in a prospective study and meta-analysis [14, 15]. Consumption of fruits and vegetables was linked to better glucose control [16]. A meta-analysis has recently shown that ultraprocessed food increases the risk of T2D [17].

However, the extent to which dietary characteristics affect glucose metabolism has yet to be entirely understood. Indeed, higher caloric intake is associated with increased blood glucose and T2D [18], but the association between the types of food consumed and blood glucose levels still needs to be determined. Furthermore, obesity status may influence the interpretation, since obesity is strongly linked to the impairment of blood glucose and the development of insulin resistance.

Therefore, we used nationally representative data from the 2018 Indonesia Basic Health Research (Riskesdas 2018/Riset Kesehatan Dasar 2018) to investigate the association between unhealthy foods and glucose metabolism. This study aimed to determine the link between several components of unhealthy foods and glucose metabolism markers in Indonesian adults with overweight or obesity.

## 2. Materials and Methods

### 2.1. Data Sources

This study used a population-based, cross-sectional, nationally representative survey (Indonesia Basic Health Research 2018/Riskesdas 2018/Riset Kesehatan Dasar 2018) conducted by the National Institute of Health Research Development (NIHRD), Ministry of Health, Indonesia. In this analysis, up to 2500 censuses from 26 provinces, including 1446 urban and 1054 rural sites, were subsampled to represent the national level of biomedical data collection. The sampling and survey methods have been described in detail [19].

Inclusion criteria of this study were as follows: individuals aged 18–50 years with a BMI of ˃23 kg/m^2^ were eligible for this study, as were those who had completed data on food frequency consumption, those who had completed data on physical activity questionnaire, and those who had completed data on fasting glucose and 2-hours postprandial glucose. Exclusion criteria of this study were as follows: respondents had a BMI ≤ 23 kg/m^2^, and the respondent was sick or did not complete all measurements during the study.

### 2.2. Measurements

Basic characteristics and anthropometric measurements (height and weight) were collected using a standardized protocol by well-trained interviewers. A multifunction brand stadiometer with a capacity of 2 m and a precision of 0.1 cm was used to measure the standing height. The body weight was measured on a Camry digital weight scale with a capacity of 150 kg. The weight scale was calibrated daily before use. The body mass index (BMI) was calculated as weight (kg)/height squared (m^2^) and was determined based on WHO criteria for the Asian population: healthy weight (18.5 to <23 kg/m^2^), overweight (23.0 to <27.5 kg/m^2^), and obese (≥27.5 kg/m^2^) [20]. Several self-reported covariates were collected through interviews: age, gender (men and women), and rural-urban living area.

The respondents were asked about the frequency of sweet, salty, high-fat, grilled, processed, and fruit and vegetable intake in the last week using a validated questionnaire and food card. Food frequency was recorded as >one time (1x)/day, 1x/day, 3–6x/week, 1-2x/week, ≤3x/month, and never. It was categorized in a binary form: frequently (≥1x/day) and rarely (<1x/day) [21]. In food questionnaires, refined carbohydrates included flour-processed foods with added sugar, such as flavored bread. Sweet foods include high-sugar foods with additional natural sugar, e. g., cakes and canned fruit. High-fat and fried foods include high-fat foods, e. g., fatty meats, oxtail soup, fried foods, foods containing coconut milk and margarine, and high-cholesterol foods, such as innards (intestines, tripe), eggs, and shrimp.

To determine blood glucose markers, fasting plasma glucose and 2-hpost-prandial glucose were employed. According to the American Diabetic Association (ADA) [22], IFG is defined as fasting blood glucose levels of 100–125 mg/dl with normal oral glucose tolerance test (OGTT) results of <140 mg/dl; IGT is defined as OGTT results of 140–199 mg/dl with normal fasting blood glucose levels of <100 mg/dl; or both IFG and IGT.

### 2.3. Statistical Analysis

Pearson's Chi-square test was used to describe the IFG/IGT status based on age groups, gender, sedentary activities, unhealthy food intake, and living area (rural or urban) as categorical variables. First, a simple regression analysis (unadjusted model) was performed with unhealthy foods (refined carbohydrates, salty food, high-fat and fried foods, grilled food, fruit, and vegetables as well as ultraprocessed foods) as independent variables, and IFG/IGT/combination between IFG and IGT as dependent variables (model 1).

Subsequently, multiple regression analysis was performed with body mass index (BMI) added as an independent variable (model 2). Next, multiple regression analysis was performed with physical activity level added as independent variables (model 3) and BMI and physical activity level added together (model 4). Finally, multiple regression analysis was performed to relate unhealthy foods (refined carbohydrates, salty food, high-fat and fried foods, grilled food, fruit, and vegetables, as well as ultraprocessed foods) and IFG/IGT or combinations of IFG and IGT adjusted by BMI, physical activity level, age, and sex (model 5). All data were analyzed using SPSS for Mac, version 22.0 (IBM Inc.), and statistical significance was set at *p* < 0.05.

### 2.4. Ethical Approval

All procedures performed in this study were in accordance with the ethical standards of the institutional research committee, the 1964 Helsinki Declaration and its later amendments, or comparable ethical standards.

## 3. Results

### 3.1. Participant Characteristics

A total of 8752 subjects were included in this study's analysis. Of these, 16.6% had IFG, 25.6% had IGT, 13.9% had combined glucose intolerance (CGI), and the rest had normal glucose regulations. The majority of individuals in the study were overweight (49.6%). Impaired glucose tolerance was the most common glucose metabolism impairment in the obese, overweight, middle-aged, young, female, urban, rural, frequent consumption of sweet foods, rare consumption of sweet foods, frequent consumption of salty foods, rare consumption of salty foods, frequent consumption of fat foods, rare consumption of fat foods, frequent consumption of grilled foods, rare consumption of grilled foods, rare consumption of processed foods, less consumption of fruit and vegetables, sufficient consumption of fruit and vegetables, lack of physical activity, and sufficient physical activity groups. Interestingly, in the male and frequently consumed processed foods subgroup, IFG was the dominant glucose metabolism disorder (Table 1).

**Figure 1:**
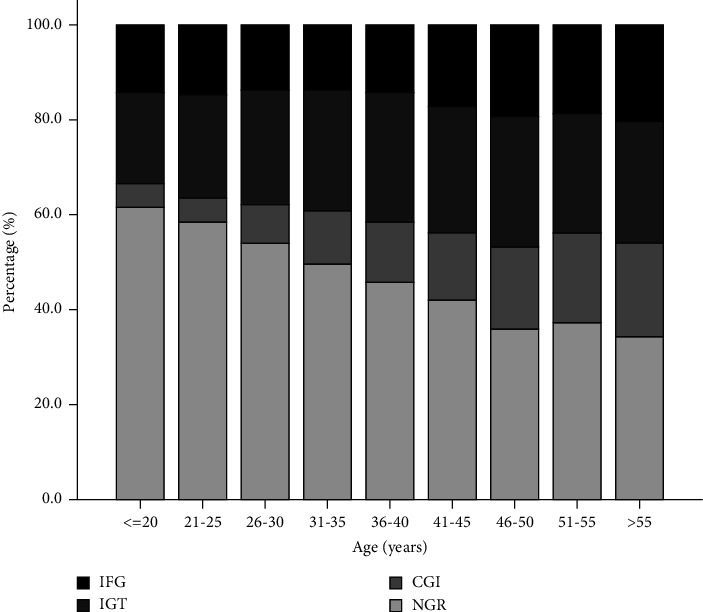
The prevalence of IFG, IGT, and CGI in participants according to different categories of age. IFG, impaired fasting glucose; IGT, impaired glucose tolerance; CGI, combined glucose intolerance; NGR, normal glucose tolerance.

Obesity affects 22.6% of the 8752 individuals in the study. In the obese group, the highest percentage of people had impaired glucose metabolism, namely IGT, CGI, and IFG (28.4%, 16.5%, and 16.3%, respectively). While in the overweight group, the highest proportions were IGT, IFG, and CGI (26.2%, 16.6%, and 14.3%, respectively) (Table 1). IFG and CGI are the most prevalent in the 46–50 year-old age group, while IGT is most common in the 41–45 year-old age group (Figure 1).

### 3.2. Association between the Dietary Patterns of Specific Food Groups and IFG

Consumption of sweet, grilled, and processed foods was significantly associated with IFG for all models tested. After adjustment for physical activity (model 2) and BMI and physical activity (model 4), frequent consumption of sweet foods had a risk of IFG of approximately 15% (OR = 1.153, 95% CI = 1.047–1.268). Frequent consumption of grilled food had higher odds of developing IFG than rare consumption of grilled food (model 2, OR = 1.350, 95% CI = 1.056–1.725). The eating pattern with the highest probability of IFG is the consumption of processed foods. In model 5, after adjusting for BMI, physical activity, age, and gender, consumption of processed food >1x/day had a risk of IFG of approximately 72% (OR = 1.729, 95% CI = 1.311–2.280) (Table 2).

### 3.3. Association between the Dietary Patterns of Specific Food Groups and IGT

The association test between sweet foods and IGT was found to have significant results in model 1 (unadjusted, *p*=0.031); however, frequent consumption of sweet foods was not a risk factor for IGT (OR = 0.924, 95% CI = 0.859–0.993). Different results were shown in the high-fat food group. Interestingly, in unadjusted model 1, consumption of high-fat foods >1x/day had the highest odds of being IGT, which were approximately 12% (OR = 1.124, 95% CI = 1.032–1.225) (Table 3).

### 3.4. Association between the Dietary Patterns of Specific Food Groups and CGI

Consumption of sweet foods was associated with CGI (model 2, *p*=0.013; model 3, *p*=0.013; model 4, *p*=0.013; model 5, *p*=0.016). In models 2, 3, and 4, frequent consumption of sweet foods had a greater probability of developing CGI (OR = 1.173, 95% CI = 1.034–1.331). All models indicate an association between processed food consumption and CGI. In model 5, after adjusting for BMI, physical activity, age, and gender, frequent consumption of processed foods had the highest odds of CGI, approximately 62% (OR = 1,620, 95% CI = 1,150–2.283) (Table 4).

## 4. Discussion

This study aimed to determine the association between unhealthy eating habits, fruit and vegetable consumption, and impaired glucose status in adults. This study's analysis revealed that eating processed foods >once per day was the strongest risk factor for IFG and CGI, whereas eating high-fat foods frequently was the highest risk factor for IGT. Consistent with the results of the present study, other investigators have reported that consuming foods rich in saturated fat and cholesterol may increase the risk of impaired glucose and insulin regulation [23, 24]. In contrast, a diet high in fruits, vegetables, and whole grains can prevent or control conditions related to insulin resistance, including IFG and IGT [24, 25].

Processed foods and high-fat foods, including fried foods, are high in salt, saturated fat, and cholesterol. The World Health Organization and most nutritional professionals today recognize that a diet rich in salt, saturated fat, and excess sugar is disease-causing. An association between a Western diet characterized by high consumption of red meat, processed meat, fast food, alcoholic beverages, and sugar-sweetened beverages and a higher risk of prediabetes has also been reported [26, 27]. A study also found that poor dietary quality, excessive consumption of cereals and salt, and insufficient consumption of vegetables, fish, and diet variety were all associated with an increased risk of prediabetes [28]. Furthermore, several studies have shown a correlation between eating green leafy vegetables (rich in vitamins, trace elements, and soluble dietary fiber) and a reduced risk of T2D [29, 30].

A healthier diet can lower the risk of the development of prediabetes into diabetes by 40% to 70% [31]. The Mediterranean and DASH (Dietary Approaches to Stop Hypertension) diets protect against the development of insulin resistance and T2D [32]. This lends credence to the theory that a plant-based diet with a balanced glycemic index and load, high in soluble fiber and phytochemicals, might be useful in lowering the risk of dysglycemia and prediabetes. The Mediterranean and DASH diets are relatively high in fat from vegetable sources (extravirgin olive oil, tree nuts). They include an abundance of minimally processed plant foods (vegetables, fruits, whole grains, and legumes), moderate fish consumption, low consumption of meat and meat products, and wine in moderation, usually with meals. It has been hypothesized that their positive impact is related to their components [33]. A biological explanation is possible. The antioxidant profile of the diet may prevent the accumulation of oxidative stress, which has been linked to the development of insulin resistance and *β*-cell dysfunction [34].

In this study, 72.17% of participants were overweight or obese; 16.53% developed IFG, 26.87% had IGT, and 14.99% developed CGI. IFG occurs due to inadequate glucose control, resulting in higher blood glucose even after an overnight fast. In contrast, IGT develops due to an individual's inability to respond to glucose taken as part of a meal, resulting in increased post-prandial blood glucose. While both IFG and IGT contribute to insulin resistance, the former is caused by hepatic insulin resistance, while the latter is caused predominantly by insulin resistance in skeletal muscle. Notably, pancreatic *β*-cell dysfunction is shared by both IFG and IGT [35, 36].

It is well established that IFG can be reverted to normal blood glucose homeostasis with effective intervention. Compared to earlier lifestyle intervention research, intensive lifestyle intervention plays a significant role in educating individuals and assisting them in achieving glycemic control [37]. Without lifestyle modifications and adequate assistance, roughly 9% of patients with IFG will acquire type 2 diabetes within three years [38]. Intensive lifestyle programs, which include diet and physical activity interventions, significantly improve fasting plasma glucose, weight, BMI, triglycerides, high-density lipoprotein cholesterol, and total cholesterol in individuals with IFG [39].

IGT is prediabetic hyperglycemia characterized by peripheral insulin resistance, and it has been demonstrated that weight loss and increases in daily energy expenditure reduce the incidence of insulin resistance [40, 41]. In IGT patients, lifestyle changes focused on physical or nutritional therapies, or both, are related to improvements in 2-hour plasma glucose and FPG levels. Furthermore, all individuals with IGT, whether they have normal or low FPG levels, may benefit from lifestyle changes to delay the development and reduce the incidence of T2DM [42, 43].

The strength of this study is that it uses a large and representative sample in Indonesia and data on dietary intake obtained through interviews using questionnaires and dietary intake cards to minimize bias. This study has limitations, including the fact that it was conducted in a cross-sectional design prospective cohort studies, or RCTs, of diet type modification and its effect on glucose control in overweight/obese people should be conducted. In addition, the data on unhealthy food intake was obtained only from frequency data, so the exact weight of the food consumed (in grams) and energy intake (in kcal) could not be determined. Finally, we do not have data on additional confounding biomarkers, such as IGF-1 levels.

## 5. Conclusions

This population-based study found that eating unhealthy diets increased the risk of impaired glucose metabolism among adults who were overweight or obese in Indonesia. Longitudinal studies should be considered to investigate the various impacts of food patterns on glucose metabolism in overweight and obese people. More importantly, health promotions on nutrition and physical activities should be encouraged among overweight and obese individuals, as they can play an essential role in developing healthy eating habits and increasing healthy living behaviors.

## Figures and Tables

**Table 1:** Characteristics of the study participants (*n* = 8752).

Characteristics	IFG (*n* = 1452)	IGT (*n* = 2243)	CGI (*n* = 1215)	NGR (*n* = 3842)
*BMI*				
Obesity, *n* (%)	1027 (16.6)	1666 (26.9)	934 (15.1)	2577 (41.5)
Overweight, *n* (%)	425 (16.7)	577 (22.6)	281 (11.0)	1265 (49.6)

*Age*				
Middle age, *n* (%)	938 (18.5)	1335 (26.3)	867 (17.1)	1929 (38.1)
Young, *n* (%)	514 (14.0)	908 (24.7)	348 (9.4)	1913 (51.9)

*Gender*				
Male, *n* (%)	497 (21.1)	454 (19.2)	300 (12.7)	1109 (47.0)
Female, *n* (%)	955 (14.9)	1789 (28.0)	915 (14.3)	2733 (42.8)

*Living area*				
Urban, *n* (%)	772 (16.8)	1063 (23.1)	648 (14.1)	2124 (46.1)
Rural, *n* (%)	680 (16.4)	1180 (28.5)	567 (13.7)	1718 (41.4)

*Sweet foods*				
Frequent, *n* (%)	509 (17.3)	682 (23.2)	446 (15.2)	1303 (44.3)
Rare, *n* (%)	943 (16.2)	1561 (26.9)	769 (13.2)	2539 (43.7)

*Salty foods*				
Frequent, *n* (%)	476 (16.3)	723 (24.8)	405 (13.9)	1317 (45.1)
Rare, *n* (%)	976 (16.7)	1520 (26.1)	810 (13.9)	2525 (43.3)

*High-fat foods*				
Frequent, *n* (%)	693 (16.2)	1136 (26.6)	614 (14.4)	1831 (42.8)
Rare, *n* (%)	759 (16.9)	1107 (24.7)	601 (13.4)	2011 (44.9)

*Grilled foods*				
Frequent, *n* (%)	60 (21.1)	63 (22.1)	45 (15.8)	117 (41.1)
Rare, *n* (%)	1392 (16.4)	2180 (25.7)	1170 (13.8)	3725 (44.0)

*Processed foods*				
Frequent, *n* (%)	48 (22.0)	45 (20.6)	43 (19.7)	82 (37.6)
Rare, *n* (%)	1404 (16.5)	2198 (25.8)	1172 (13.7)	3760 (44.1)

*Fruits and vegetables*				
Deficient, *n* (%)	1340 (16.8)	2025 (25.4)	1113 (13.9)	3504 (43.9)
Sufficient, *n* (%)	112 (14.5)	218 (28.3)	102 (13.2)	338 (43.9)

*Physical activities*				
Deficient, *n* (%)	207 (19.9)	232 (22.3)	133 (12.8)	470 (45.1)
Sufficient, *n* (%)	1245 (16.1)	2011 (26.1)	1082 (14.0)	3372 (43.7)

IFG, impaired fasting glucose; IGT, impaired glucose tolerance; CGI, combined glucose impairment; NGR, normal glucose regulation; BMI, body mass index; MET, metabolic equivalent of task; frequent, ≥1x/day; rare, <1x/day; deficient, <5 potion/day or <150 minutes/week; sufficient, ≥5 portion/day or >150 minutes/week.

**Table 2:** There is an association between the dietary patterns of specific food groups and IFG.

Food groups	Model 1^a^			Model 2^b^			Model 3^c^			Model 4^d^			Model 5^e^		
	OR	95% CI	*p*	OR	95% CI	*p*	OR	95% CI	*p*	OR	95% CI	*p*	OR	95% CI	*p*
*Sweet foods*															
Rare	1			1			1			1			1		
Frequent	1.099	1.019–1.186	0.015^*∗*^	1.153	1.047–1.268	0.004^*∗*^	1.152	1.047–1.268	0.004^*∗*^	1.153	1.047–1.268	0.004^*∗*^	1.148	1.042–1.264	0.005^*∗*^

*Salty foods*															
Rare	1			1			1			1			1		
Frequent	0.978	0.888–1.077	0.653	0.978	0.888–1.078	0.657	0.978	0.888–1.078	0.657	0.979	0.888–1.078	0.660	1.003	0.909–1.106	0.960

*High-fat foods*															
Rare	1			1			1			1			1		
Frequent	1.010	0.922–1.106	0.831	1.007	0.919–1.103	0.883	1.011	0.923–1.107	0.814	1.008	0.920–1.104	0.866	1.024	0.933–1.122	0.621

*Grilled foods*															
Rare	1			1			1			1			1		
Frequent	1.344	1.052–1.718	0.018^*∗*^	1.350	1.056–1.725	0.017^*∗*^	1.337	1.046–1.708	0.020^*∗*^	1.342	1.050–1.716	0.019^*∗*^	1.347	1.051–1.727	0.019^*∗*^

*Processed foods*															
Rare	1			1			1			1			1		
Frequent	1.657	1.261–2.178	≤0.001^*∗*^	1.650	1.255–2.169	≤0.001^*∗*^	1.655	1.259–2.175	≤0.001^*∗*^	1.648	1.254–2.166	≤0.001^*∗*^	1.729	1.311–2.280	≤0.001^*∗*^

*Fruits and vegetables*															
Sufficient	1			1			1			1			1		
Deficient	1.153	0.978–1.359	0.091	1.142	0.968–1.346	0.115	1.147	0.973–1.353	0.102	1.136	0.964–1.340	0.129	1.146	0.970–1.354	0.109

^a^Unadjusted; ^b^adjusted for BMI; ^c^adjusted for physical activity; ^d^adjusted for BMI and physical activity; ^e^adjusted for BMI, physical activity, age, and gender; ^*∗*^significant, *p* < 0.05.

**Table 3:** There is an association between the dietary patterns of specific food groups and IGT.

Food groups	Model 1^a^			Model 2^b^			Model 3^c^			Model 4^d^			Model 5^e^		
	OR	95% CI	*p*	OR	95% CI	*p*	OR	95% CI	*p*	OR	95% CI	*p*	OR	95% CI	*p*
*Sweet foods*															
Rare	1			1			1			1			1		
Frequent	0.924	0.859–0.993	0.031^*∗*^	0.930	0.849–1.019	0.120	0.930	0.849–1.019	0.120	0.930	0.849–1.019	0.120	0.927	0.846–1.016	0.107

*Salty foods*															
Rare	1			1			1			1			1		
Frequent	0.945	0.863–1.035	0226	0.945	0.863–1.036	0.228	0.945	0.863–1.035	0.222	0.945	0.862–1.035	0.224	0.940	0.857–1.031	0.187

*High-fat foods*															
Rare	1			1			1			1			1		
Frequent	1.124	1.032–1.225	0.007^*∗*^	1.120	1.027–1.220	0.010^*∗*^	1.122	1.030–1.223	0.008^*∗*^	1.117	1.025–1.218	0.011^*∗*^	1.111	1.018–1.211	0.018^*∗*^

*Grilled foods*															
Rare	1			1			1			1			1		
Frequent	0.932	0.731–1.189	0.570	0.937	0.734–1.196	0.601	0.943	0.739–1.203	0.635	0.948	0.742–1.210	0.668	0.965	0.754–1.235	0.779

*Processed foods*															
Rare	1			1			1			1			1		
Frequent	1.037	0.789–1.364	0.793	1.029	0.782–1.354	0.840	1.041	0.791–1.369	0.777	1.032	0.784–1.358	0.823	1.068	0.809–1.409	0.644

*Fruits and vegetables*															
Sufficient	1			1			1			1			1		
Deficient	0.911	0.784–1.059	0.224	0.896	0.771–1.042	0.154	0.919	0.791–1.068	0.271	0.904	0.778–1.051	1.051	0.898	0.771–1.045	0.165

^a^Unadjusted; ^b^adjusted for BMI; ^c^adjusted for physical activity; ^d^adjusted for BMI and physical activity; ^e^adjusted for BMI, physical activity, age, and gender; ^*∗*^significant, *p* < 0.05.

**Table 4:** There is an association between the dietary patterns of specific food groups and CGI.

Food groups	Model 1^a^			Model 2^b^			Model 3^c^			Model 4^d^			Model 5^e^		
	OR	95% CI	*p*	OR	95% CI	*p*	OR	95% CI	*p*	OR	95% CI	*p*	OR	95% CI	*p*
*Sweet foods*															
Rare	1			1			1			1			1		
Frequent	1.067	0.961–1.184	0.225	1.173	1.034–1.331	0.013^*∗*^	1.173	1.034–1.331	0.013^*∗*^	1.173	1.034–1.331	0.013^*∗*^	1.169	1.030–1.328	0.016^*∗*^

*Salty foods*															
Rare	1			1			1			1			1		
Frequent	0.998	0.877–1.135	0.973	0.998	0.878–1.135	0.979	0.998	0.971–1.134	0.971	0.998	0.878–1.135	0.976	1.012	0.889–1.152	0.855

*High-fat foods*															
Rare	1			1			1			1			1		
Frequent	1.082	0.959	0.201	1.078	0.954–1.217	0.228	1.081	0.958–1.221	0.207	1.076	0.953–1.215	0.234	1.084	0.959–1.224	0.198

*Grilled foods*															
Rare	1			1			1			1			1		
Frequent	1.169	0.845–1.617	0.344	1.175	0.849–1.625	0.332	1.176	0.850–1.627	0.326	1.181	0.854–1.635	0.315	1.196	0.862–1.659	0.285

*Processed foods*															
Rare	1			1			1			1			1		
Frequent	1.543	1.099–2.167	0.012^*∗*^	1.533	1.091–2.153	0.014^*∗*^	1.546	1.101–2.170	0.012^*∗*^	1.535	1.093–2.156	0.013^*∗*^	1.620	1.150–2.283	0.006^*∗*^

*Fruits and vegetables*															
Sufficient	1			1			1			1			1		
Deficient	1.061	0.853–1.319	0.593	1.045	0.840–1.299	0.694	1.066	0.857–1.326	0.565	1.050	0.844–1.306	0.664	1.051	0.844–1.309	0.655

^a^Unadjusted; ^b^adjusted for BMI; ^c^adjusted for physical activity; ^d^adjusted for BMI and physical activity; ^e^adjusted for BMI, physical activity, age, and gender; ^*∗*^significant, *p* < 0.05.

## Data Availability

The data used to support this study are available from the Data Management Laboratory of NIHRD, the Ministry of Health, and the Republic of Indonesia on reasonable request with prior officially written permission.
